# Mixture of Expert-Based SoftMax-Weighted Box Fusion for Robust Lesion Detection in Ultrasound Imaging

**DOI:** 10.3390/diagnostics15050588

**Published:** 2025-02-28

**Authors:** Se-Yeol Rhyou, Minyung Yu, Jae-Chern Yoo

**Affiliations:** Department of Electrical and Computer Engineering, College of Information and Communication Engineering, Sungkyunkwan University, Suwon 440-746, Republic of Korea; fbtpduf@naver.com (S.-Y.R.); 6unhuiw@gmail.com (M.Y.)

**Keywords:** hepatocellular carcinoma, ultrasound, SoftMax-weighted box fusion, clustering, CNN, deep learning

## Abstract

**Background/Objectives:** Ultrasound (US) imaging plays a crucial role in the early detection and treatment of hepatocellular carcinoma (HCC). However, challenges such as speckle noise, low contrast, and diverse lesion morphology hinder its diagnostic accuracy. **Methods:** To address these issues, we propose CSM-FusionNet, a novel framework that integrates clustering, SoftMax-weighted Box Fusion (SM-WBF), and padding. Using raw US images from a leading hospital, Samsung Medical Center (SMC), we applied intensity adjustment, adaptive histogram equalization, low-pass, and high-pass filters to reduce noise and enhance resolution. Data augmentation generated ten images per one raw US image, allowing the training of 10 YOLOv8 networks. The mAP@0.5 of each network was used as SoftMax-derived weights in SM-WBF. Threshold-lowered bounding boxes were clustered using Density-Based Spatial Clustering of Applications with Noise (DBSCAN), and outliers were managed within clusters. SM-WBF reduced redundant boxes, and padding enriched features, improving classification accuracy. **Results:** The accuracy improved from 82.48% to 97.58% with sensitivity reaching 100%. The framework increased lesion detection accuracy from 56.11% to 95.56% after clustering and SM-WBF. **Conclusions:** CSM-FusionNet demonstrates the potential to significantly improve diagnostic reliability in US-based lesion detection, aiding precise clinical decision-making.

## 1. Introduction

Liver cancer is a major cause of cancer-related mortality [1,2,3], ranking as the third leading cause of cancer deaths worldwide in 2022, with 865,000 new cases and 757,948 deaths reported [4]. Particularly, primary liver cancer is predominantly composed of HCC [5], which accounts for 75–85% of cases, and intrahepatic cholangiocarcinoma, representing 10–15%. Globally, chronic infection with HBV or HCV is associated with 21% to 55% of HCC cases [6,7]. The high mortality rate of liver cancer can be attributed to its late-stage detection, which significantly limits therapeutic options and reduces the likelihood of favorable outcomes [8,9]. Luckily, early identification of HCC allows for surgical resection, providing a favorable prognosis, with 5-year survival rates exceeding 70% [10,11]. Therefore, regular HCC screening is essential for individuals at risk to ensure early detection of HCC [12,13,14].

Among screening methods for HCC, ultrasonography is a non-invasive, cost-effective, and widely accessible imaging technique that provides real-time liver visualization without ionizing radiation [15]. These attributes make it ideal for regular screening and surveillance in high-risk populations, particularly in low- and middle-income countries where liver cancer incidence is disproportionately high [16,17]. Despite its advantages, the efficacy of US depends on the operator’s expertise and the quality of the equipment [18,19,20,21]. Additionally, the detection of small lesions (<2 cm) remains challenging, necessitating complementary methods such as serum biomarkers or advanced imaging techniques for confirmatory diagnosis [22].

Nevertheless, combining ultrasonography with other non-invasive approaches holds significant potential for improving the early detection of liver cancer. Advanced imaging techniques are currently being developed to improve the detectability of HCC and enhance the characterization of HCC nodules [23,24,25]. Recently, emerging artificial intelligence technologies, featuring powerful brain-like algorithms in the field of medical imaging, have significantly contributed to improving the accuracy of diagnoses [13,14,15,16,17,18,19]. Numerous artificial intelligence approaches based on deep learning have been proposed to address the challenges [26,27,28,29,30,31,32] and issues related to ultrasonography diagnostic accuracy of HCC as well as the expertise required [33,34,35,36,37].

Ryu et al. (2021) [33] proposed a joint segmentation and classification system for hepatic lesions in ultrasound images, utilizing a shared encoder with two branches. The input combined a grayscale image with Euclidean distance maps of foreground and background clicks provided by the user. The system achieved 89.8% accuracy for classification and 90.4% accuracy for joint segmentation and classification. The segmentation network employed bilinear interpolation for upsampling, reducing parameters and mitigating overfitting compared to FCNs. The classification branch, based on a VGG-16 architecture, predicted lesion types from shared convolutional features. While the integration of segmentation and classification demonstrated potential, the performance exhibited limitations, particularly in fully leveraging joint learning. The approach highlights the challenges of combining these tasks effectively in medical imaging.

Zhao et al. (2023) [34] developed a lightweight neural network, USC-Enet, designed for small-scale medical image datasets, incorporating an attention mechanism to address overfitting issues common with small datasets. Using 2168 images, the model achieved a sensitivity of 0.915. To improve performance, the study streamlined the network structure for transfer learning, reduced network parameters to prevent overfitting, and combined image features with clinical data using a random forest classifier. This end-to-end model integrated Convolutional Neural Network (CNN) feature maps with clinical data, such as age, gender, hepatitis history, and tumor markers like alpha-fetoprotein, but sensitivity score remains suboptimal.

Poreddy et al. (2024) [35] proposed a classification model for focal liver lesions by applying the discrete Haar wavelet transform to each frame of a focal liver lesions video, decomposing each frame into multiple subbands. Singular value decomposition was performed on each subband, and the maximum value among the columns of the Singular Value Decomposition matrices was extracted as a frame-level statistical feature. These features were averaged across all video frames and fed into a decision tree classifier. Experimental results achieved an accuracy of 97.18%, surpassing conventional methods. The authors chose the decision tree due to its robustness on small datasets and ability to capture simple relationships without overfitting, leading to superior performance compared to other classifiers.

Chaiteerakij et al. (2024) [36], in a retrospective study using 26,288 ultrasound images from 5444 patients, developed and evaluated an AI-assisted system for detecting and classifying seven types of focal liver lesions, including HCC. They employed YOLOv5, which predicts multiple bounding boxes per grid cell to handle objects of varying sizes and aspect ratios. To remove duplicates, non-maximum suppression was applied, retaining only the bounding box with the highest confidence score in each group. By integrating YOLOv5 with the Darknet architecture and Cross-Stage Hierarchical Networks (CSPNet), and pretraining on the COCO dataset, the system achieved an 84% detection rate for HCC (among all lesions) and an overall accuracy of 94%. Despite its high performance, the model’s computational complexity may limit its adoption in healthcare settings where computing resources are constrained.

In this study, we present CSM-FusionNet, a novel deep learning-based fusion model designed to integrate Clustering, SoftMax-weighted Box Fusion, and Mixture of Experts. The proposed framework is structured around four core functionalities, each contributing distinct advantages to the overall system. A concise description of these components is provided below to highlight their specific roles and significance within the network.

1. Image Preprocessing: Due to the inherent characteristics of US images, significant speckle noise and variations in resolution arise depending on device settings. To address these issues, four distinct image processing filters were applied to reduce noise and accommodate diverse resolutions. This approach also facilitated data augmentation, expanding a single US image into ten variations for enhanced training and analysis.
2. Lesion Detection with YOLOv8: To detect lesions within the liver region, we utilized YOLOv8, one of the most widely adopted object detection models. By leveraging the ten augmented US images, we constructed ten individual object detection networks. The mAP@0.5 of each network was measured to serve as weights for the subsequent bounding box fusion process.
3. Bounding Box Optimization: This stage represents the most technically complex and critical component of the framework. A low detection threshold is applied across the ten networks to generate multiple bounding boxes for regions suspected of containing lesions. Clustering is then performed on the resulting bounding boxes to identify distinct lesion-distributed regions. Subsequently, these multiple boxes are consolidated into a single representative box for each region using SM-WBF, an adaptation of the Weighted Box Fusion (WBF) method tailored to our approach. To mitigate potential information loss during the fusion process, padding is applied to each final bounding box as a concluding step.
4. Lesion Classification: The bounding boxes generated through the optimization process are ultimately classified into three categories: benign, malignant, and error. Each US image may yield one or multiple lesion boxes, all of which are subjected to classification. Among the classified results, boxes labeled as “error” are disregarded. In cases where both benign and malignant classifications are present within the same image, the lesion is conservatively categorized as malignant to prioritize diagnostic sensitivity.

Our technology holds the potential to achieve high accuracy and efficiency in the detection and analysis of lesions using ultrasound imaging. By employing advanced fusion techniques, such as SM-WBF and clustering algorithms, the system integrates information from multiple networks to deliver reliable diagnostic results. This enables healthcare professionals to detect even subtle lesions, reduces diagnostic time, and supports optimal decision-making for personalized treatment. Furthermore, the automated nature of the technology enhances resource efficiency and enables precise diagnostics even in regions with limited medical infrastructure, thereby improving healthcare accessibility. Ultimately, this innovation emphasizes the importance of early diagnosis and preventive care, contributing to increased patient survival rates and improved quality of life.

## 2. Materials and Methods

### 2.1. Dataset Preparation

**Figure 1:**
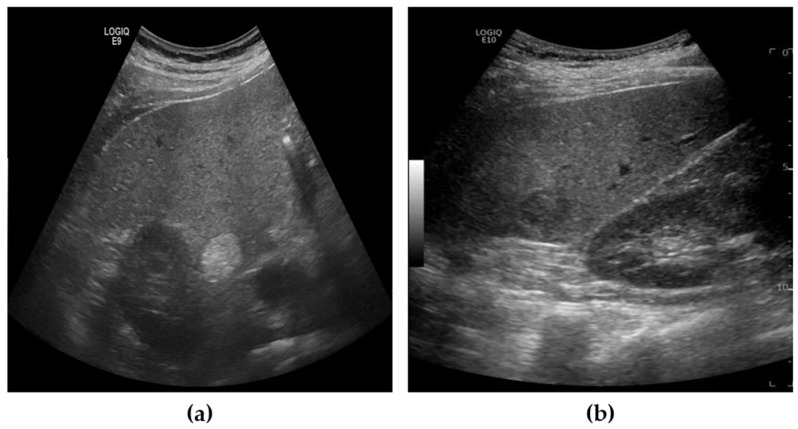
Example of US images from dataset. (**a**) Benign, (**b**) Malignant.

The liver US images used in this study were acquired using the Siemens ACUSON Sequoia 512 system (Siemens Healthineers, Erlangen, Germany), which operates at a frequency range of 3 to6 MHz, with 256 gray levels and a maximum depth of 36 cm. These images were collected under data use agreements and approved by the Institutional Review Board (IRB) of SMC, one of South Korea’s leading hospitals (SMC-2020-10-178-002). The dataset was annotated with two categories: benign and malignant. Since accurate diagnosis using only US images is challenging, the final labels were determined by SMC’s expert physicians after completing all necessary diagnostic procedures, including CT, MRI, and biopsy. The annotation for each patient’s US images was based on these comprehensive findings. Given the goal of our study to develop a screening tool, lesions were categorized simply into benign or malignant, rather than employing more granular labeling. Figure 1 provides examples of benign and malignant cases from our dataset.

The dataset consisted of a total of 1054 ultrasound images, collected from patients aged 48 to 82 years. Among these, 746 images were labeled as benign, and 308 images were labeled as malignant. For model development and evaluation, the dataset was divided into training, validation, and test sets in a 6:2:2 ratio.

### 2.2. Proposed Deep Learning Neural Network: CSM-FusionNet

**Figure 2:**
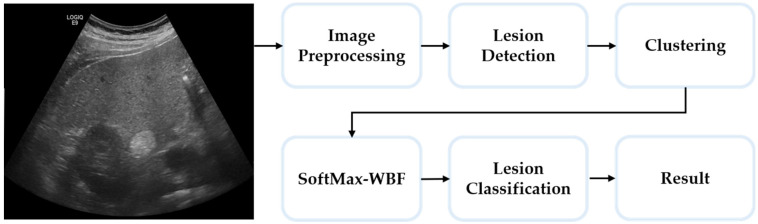
Flowchart of our proposed network: CSM-FusionNet.

As illustrated in Figure 2, this flowchart outlines the framework of our proposed network, CSM-FusionNet. The input images are US images collected from SMC. These images undergo preprocessing to address the dynamic options and characteristics of US equipment. To this end, we applied a combination of filters, including a low-pass Gaussian filter, a high-pass sharpening spatial filter, an intensity adjustment filter, and an adaptive histogram equalization filter. By combining these filters, each original image was augmented into ten variations, which were then processed through ten distinct object detection models to identify potential lesions. The resulting detections produced numerous bounding boxes, requiring an ensemble approach to identify the true lesion locations. To achieve this, we utilized DBSCAN, a clustering algorithm, and a gate network inspired by Mixture of Experts, to perform SM-WBF. This process refined the bounding boxes to isolate regions most likely to contain actual lesions. During clustering, bounding boxes significantly larger or smaller than the average size of their respective clusters were filtered out as outliers. The detected lesions were then classified using a CNN, categorizing each into benign, malignant, or error classes. The error class was specifically introduced to exclude incorrectly detected lesions. This comprehensive approach allows the system to effectively handle the inherent diversity of US images while maintaining high diagnostic accuracy.

Section 2.3 describes the method for generating ten augmented images from a single ultrasound image using a combination of preprocessing filters. In Section 2.4, the process of lesion detection is outlined, where each of the ten augmented images is analyzed using individual object detection models, and the mAP@0.5 values for each network are calculated. Section 2.5 explains the normalization of weights based on the mAP@0.5 values and introduces the clustering-based SM-WBF method for optimizing bounding boxes. Finally, Section 2.6 details the lesion classification process, where the refined bounding boxes are categorized into benign, malignant, or error classes.

### 2.3. Dataset Preprocessing

**Figure 3:**
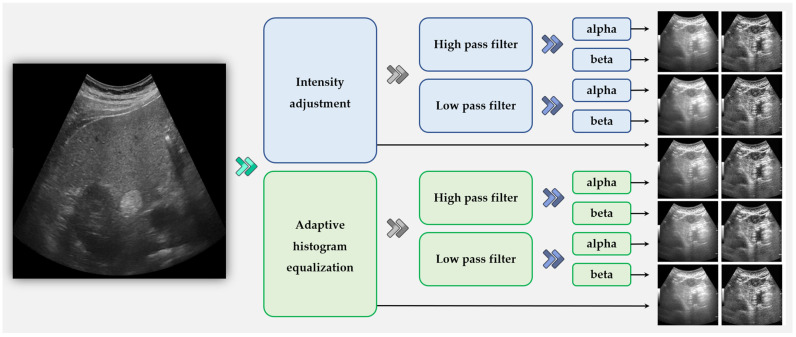
The original US image serves as the input image, and through the application of four distinct filters combined with alpha and beta values, a total of ten augmented images are generated.

The preprocessing of US images is the first step in our framework. Due to the inherent characteristics of ultrasound equipment, US images often contain noise and exhibit varying resolutions depending on dynamic settings. To generalize these differences, we applied multiple filters to augment the images. A total of four filters were used, with alpha and beta values incorporated for preprocessing. First, a contrast enhancement process was applied using the intensity adjustment filter and adaptive histogram equalization filter, generating two images from the original US image. Additionally, low-pass and high-pass filters were applied, with variations introduced through alpha and beta values, resulting in two distinct images for each filter type. By combining these four filters, a total of ten augmented images were generated. Further details of this process are illustrated in Figure 3.

As shown in Figure 3, the images processed with the high-pass filter appeared significantly sharper, while those processed with the low-pass filter exhibited a more blurred effect compared to the original image. This approach allowed us to effectively handle variations in resolution and depth differences inherent in ultrasound imaging. The alpha and beta values used for preprocessing were set empirically, with 1.5 and 0.5 selected during our experiments.

### 2.4. Lesion Detect with YOLOv8

**Figure 4:**
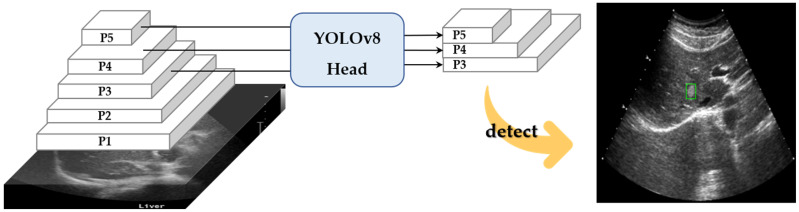
Lesion suspected regions, green bounding box, detected by YOLOv8.

Since ultrasound imaging is frequently used for real-time diagnostics, fast lesion detection is crucial. YOLOv8 offers a more lightweight architecture and optimized computation compared to YOLOv5, enabling faster inference speed. For this reason, we chose YOLOv8 for our approach. As illustrated in Figure 4, we employed YOLOv8 to develop the lesion detector and trained a total of ten networks using ten different datasets. A low threshold was applied during the detection process to generate a larger number of bounding boxes. While a higher threshold might improve accuracy metrics, it often results in missing smaller lesions, particularly those in early stages, or lesions that deviate significantly from the average size. Such omissions would render the system less effective in clinical applications, despite high accuracy metrics. By applying a lower threshold, each network produced between zero and five bounding boxes, which were then utilized in the subsequent steps.

### 2.5. Bounding Box Optimization by Clustering, SM-WBF and Padding

In Section 2.4, numerous bounding boxes are generated by ten YOLOv8 networks. These bounding boxes may focus on a single location, concentrate on multiple areas, or, in some cases, fail to detect any region. To address all these scenarios, clustering was employed to identify regions where bounding boxes are concentrated, followed by an ensemble process to consolidate multiple boxes into a single representative box for each region. The detailed methodology is outlined as follows.

#### 2.5.1. Clustering Using DBSCAN

When utilizing multiple networks for bounding box detection, issues arise where bounding boxes for the same object may be generated in slightly different locations or where multiple overlapping boxes are created. In this study, ten networks were employed, and a low threshold was set to enhance detection sensitivity. This approach resulted in a significant number of overlapping bounding boxes. To address this, DBSCAN was applied for clustering. DBSCAN is a density-based clustering algorithm that groups closely located bounding boxes into clusters based on their center coordinates (*x_center_*, *y_center_*). Bounding boxes that are sufficiently close are assigned to the same cluster, while in traditional DBSCAN, boxes in sparsely populated areas would be considered noise. However, in this study, such boxes are treated as important data and reassigned to new clusters. For clustering, the distance criterion (*eps*) was set to 0.1, and the minimum number of samples was set to 1 to ensure that at least one bounding box could form a cluster.

As a result, each cluster contained between one and five bounding boxes. Unlike traditional DBSCAN, all bounding boxes, including those that might be classified as noise, were retained as clusters for further analysis. The clustered results were then passed to the next stage of the process.

#### 2.5.2. Calculate SoftMax Weights Based on the mAP@0.5 Score

To integrate the bounding box detection results, this study employed a Mixture of Experts (MoE) approach to calculate weights that reflect the performance of each network. MoE combines the outputs of multiple expert models to produce optimal results, with a mechanism that dynamically adjusts the contribution of each expert. The performance of each expert is evaluated based on its reliability and relevance to the problem, maximizing the quality of the outcome.

In this study, the metric used to quantify the importance of each expert was the mAP@0.5 (Mean Average Precision at Intersection of Union (IoU) threshold 0.5) of each network. This metric provides a numerical representation of detection performance, where a higher mAP value indicates more reliable results. However, directly using mAP values does not effectively capture the non-linear differences in performance across networks. To address this, SoftMax normalization was applied to dynamically compute the relative importance of each network.

SoftMax normalization is closely aligned with the expert selection mechanism in MoE. By reflecting performance differences among networks, it ensures that higher-performing networks contribute more significantly to the final detection results. This approach effectively implements the principles of MoE, leveraging SoftMax to prioritize networks that generate more accurate and reliable predictions.

i. Performance-based weight assignment reflects the relative performance differences among networks, ensuring that networks with higher mAP values are assigned greater importance.
ii. Expressing normalized contribution normalizes the weights of all networks, clearly illustrating the relative contributions of each network to the outcome.
iii. Non-linear influence enhancement amplifies the contribution of high-performing networks non-linearly as the performance gap increases, ensuring a stronger influence from superior networks.

The SoftMax weights calculated in this manner are applied to the bounding box detection integration process based on the expert selection principle of the MoE. SoftMax normalization is defined by the following equation:

(1) $${SoftMax\text{\_}w}_{i}=\frac{e^{{mAP}_{i}}}{\sum_{j=1}^{N}e^{{mAP}_{j}}}$$

where *N* is the total number of YOLO networks.

#### 2.5.3. Filter out Outlier Bounding Boxes

We employed ten networks and lowered the detection threshold to identify all regions suspected of being lesions. As a result, the bounding box detection outputs may include outliers that are either excessively large or small. Such outliers significantly deviate from the average size of their respective clusters and can reduce the reliability of the results. To address this, the bounding boxes within each cluster were refined based on their dimensions, removing those identified as outliers before proceeding to the integration process. Outlier removal was performed using the width and height of the bounding boxes within each cluster. Bounding boxes that deviated beyond a predefined outlier threshold from the average size were excluded. This process minimized the impact of extreme values on the subsequent SoftMax-weighted box fusion, thereby improving the quality of the final bounding boxes. Specifically, the average width and height of the bounding boxes in each cluster were defined as $\bar{w}$ and $\bar{h}$, respectively. A bounding box with width *w* and height *h* was considered valid if it satisfied the following conditions:

(2) $$\left(1-threshold\right)\times \bar{w}\leq w<\left(1+threshold\right)\times \bar{w},\;(1-threshold)\times \bar{h}\leq h<(1+threshold)\times \bar{h}$$

where the threshold represents the allowable deviation ratio, set to 0.5 in this study.

Through this process, outliers were removed from all bounding boxes within each cluster, and the refined data were used in the subsequent integration stage. This outlier removal step is essential to ensure the reliability and accuracy of the results by preventing size distortions in bounding boxes and producing more consistent outcomes.

#### 2.5.4. Bounding Box Fusion with SM-WBF

After clustering the bounding box detection results and removing outlier boxes, SM-WBF is applied to generate a single representative box for each cluster. This method focuses on merging the bounding boxes within each cluster identified through DBSCAN in Section 2.5.1, removing redundancies, and producing an optimal result. SM-WBF specifically incorporates the SoftMax weights calculated in the earlier stage to implement a fusion strategy that reflects the performance of each network.

SM-WBF computes the weighted average of the center coordinates (*x_center_*, *y_center_*) and the dimensions (width *w*, height *h*) of each bounding box, using the SoftMax weights. This ensures that the detection results from networks with higher mAP values have a greater influence on the final box. By incorporating SoftMax normalization, the relative importance of all networks is considered, closely aligning with the expert selection mechanism of MoE. Unlike traditional WBF methods, which rely on confidence scores, our approach treats all detected bounding boxes as equally valid, excluding confidence scores from the fusion process. During the box integration process, the coordinates and dimensions of the boxes within each cluster are computed using the following equations:

(3) $${\hat{x}}_{center}=\frac{\sum_{i=1}^{N}{SoftMax\text{\_}w}_{i}\times x_{center,i}}{\sum_{i=1}^{N}{SoftMax\text{\_}w}_{i}},{\hat{y}}_{center}=\frac{\sum_{i=1}^{N}{SoftMax\text{\_}w}_{i}\times y_{center,i}}{\sum_{i=1}^{N}{SoftMax\text{\_}w}_{i}}\;\hat{width}=\frac{\sum_{i=1}^{N}{SoftMax\text{\_}w}_{i}\times {width}_{i}}{\sum_{i=1}^{N}{SoftMax\text{\_}w}_{i}},\hat{height}=\frac{\sum_{i=1}^{N}{SoftMax\text{\_}w}_{i}\times {height}_{i}}{\sum_{i=1}^{N}{SoftMax\text{\_}w}_{i}}$$

where ${\hat{x}}_{center}$ and ${\hat{y}}_{center}$ represent the center coordinates of the integrated representative bounding box, and $\hat{width}$ and $\hat{height}$ denote the width and height of the integrated box. N is the number of bounding boxes within the cluster. ${SoftMax\text{\_}w}_{i}$ is the SoftMax weight calculated in the previous step, and *x_center_*, *y_center_*, *width_i_*, *height_i_* are the coordinates and dimensions of the *i*-th bounding box within the cluster.

The key innovation of the proposed SM-WBF method lies in the application of mAP-based SoftMax weights, ensuring that the results from higher-performing networks contribute more significantly to the final bounding box computation. This approach minimizes the influence of incorrect detections from lower-performing networks while optimizing the result by comprehensively integrating the information from all bounding boxes within a cluster. By incorporating the principles of MoE, the method bases its final decisions on the relative reliability of each expert model (network), thereby enhancing the overall accuracy of the results. The representative bounding box generated through this process is subsequently used in the next stages, including outlier removal and padding application, to further refine the detection outcome.

#### 2.5.5. Add Padding to Bounding Boxes

In ultrasound imaging, the contrast between the tumor contour and the surrounding liver tissue provides critical clinical information. Therefore, preserving the detected tumor boundary is essential for accurate analysis and diagnosis. However, during the process of generating bounding boxes through clustering, there is a risk of partially cropping the tumor boundary, which can undermine the reliability of the detection results. To prevent this and retain additional information, such as color contrast around the tumor, padding was applied to the bounding boxes.

Padding involves extending the boundaries of the bounding box to include the detected object and its surrounding area. The padding size was defined in pixel units, and the YOLO-format bounding boxes were converted into pixel coordinates to facilitate the process. After extending each boundary by a specified number of pixels, the padded bounding box was converted back to the YOLO format to maintain compatibility with the detection system. Padding was applied using the following formula:

(4) $$x_{left}=x_{min}-p,\;x_{right}=x_{max}+p,\;y_{left}=y_{min}-p,\;y_{left}=y_{max}+p$$

where $x_{min},\;x_{max},\;y_{min},\;y_{max}$ represent the original bounding box coordinates, and *p* denotes the number of pixels added to each boundary.

The padding size was optimized based on the characteristics of ultrasound imaging and the requirements for tumor analysis. By applying padding, the bounding box encompassed not only the detected object’s boundaries but also its surrounding tissue. This approach preserved essential information for analyzing the contrast between the tumor boundary and the liver tissue, preventing boundary loss during the clustering process. As a result, the application of padding significantly improved the reliability of bounding box-based detection.

**Figure 5:**
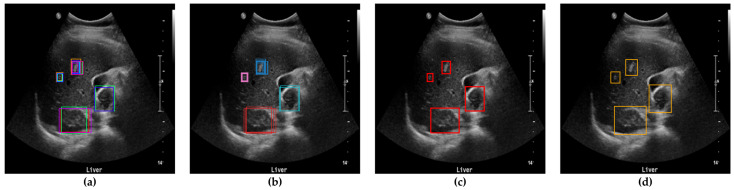
Determination of bounding boxes using clustering, SM-WBF, and padding. (**a**) All bounding boxes detected by the ten networks. (**b**) Clustering of bounding boxes into four regions using DBSCAN. (**c**) Application of SM-WBF with SoftMax weights to the clustered regions. (**d**) Addition of padding to the bounding boxes in (**c**).

**Figure 6:**
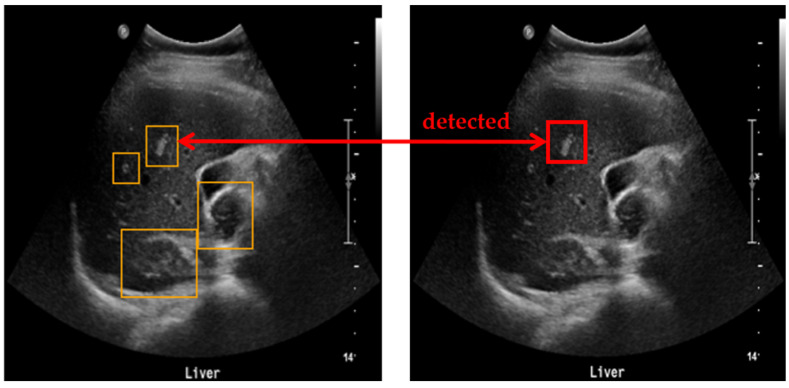
Method for measuring lesion detection accuracy. Among the four detected bounding boxes, at least one box has an IoU of 0.9 or higher with the ground truth, and, therefore, the detection is considered successful.

To evaluate the accuracy of lesion detection after applying clustering, SM-WBF, and padding, a novel measurement method was introduced. The primary goal was to ensure that all regions suspected of being lesions were identified, minimizing the chances of missing any lesions. As illustrated in Figure 5, all detected lesion-suspected regions were compared with the ground truth, and if at least one box matched, the detection was considered successful. A match was determined by comparing the IoU between the detected box and the ground truth box including padding. An IoU of 0.9 or higher was used as the threshold for a match, ensuring high precision in detection evaluation (Figure 6).

### 2.6. Lesion Classification

The final step is lesion classification. Up to this point, multiple processes such as clustering and SM-WBF were employed to accurately locate and classify the lesions. The lesion classification model provides predictions for the class of each detected lesion through YOLO, and this information supports clinical decision-making. The lesions were categorized into three classes: benign, malignant, and error.

The inclusion of the error class was essential due to the lowered detection threshold used during the YOLO process, which aimed to detect all potentially suspicious lesions. After the clustering and SM-WBF processes reduced the bounding boxes to a single box per region, the classification output for that box became the final result. In cases where more than one bounding box remained, the following criteria were applied: if all bounding boxes, excluding the error class, were classified as benign, the final result was benign; however, if even one bounding box was classified as malignant, the result was determined to be malignant. This decision-making process ensured that any potentially malignant lesions were not overlooked, thereby increasing sensitivity and enhancing the clinical value of the proposed approach.

## 3. Result

Our proposed CSM-FusionNet was implemented using MATLAB R2023b on a computer equipped with a GeForce RTX 3090 GPU with 24 GB of memory. Liver ultrasound images, annotated by experts, were collected from Samsung Medical Center to evaluate the performance of the proposed model. As demonstrated in the following experimental results, CSM-FusionNet effectively reduced false detections caused by variations in lesion size and other noise factors. Moreover, it addressed the limitations of traditional confidence-based WBF by introducing the novel SM-WBF method. Detailed quantitative results are provided below.

### 3.1. Result of Bounding Box Optimization by Clustering, SM-WBF and Padding

In this study, clustering, SM-WBF, and padding were applied to optimize bounding boxes. This optimization process aimed to remove redundancy among detected bounding boxes, refine outliers, and preserve the contour information of lesions, thereby enhancing the reliability and accuracy of the final detection results. Using DBSCAN, bounding boxes detecting the same lesion were grouped into clusters. Subsequently, bounding boxes within each cluster were refined by identifying outliers based on their dimensions $\hat{width}$ and $\hat{height}$. Boxes deviating more than 50% from the average size of the cluster were considered outliers and reduced. This step was essential to prevent distortion in cluster size and ensure stability during the integration process.

**Figure 7:**
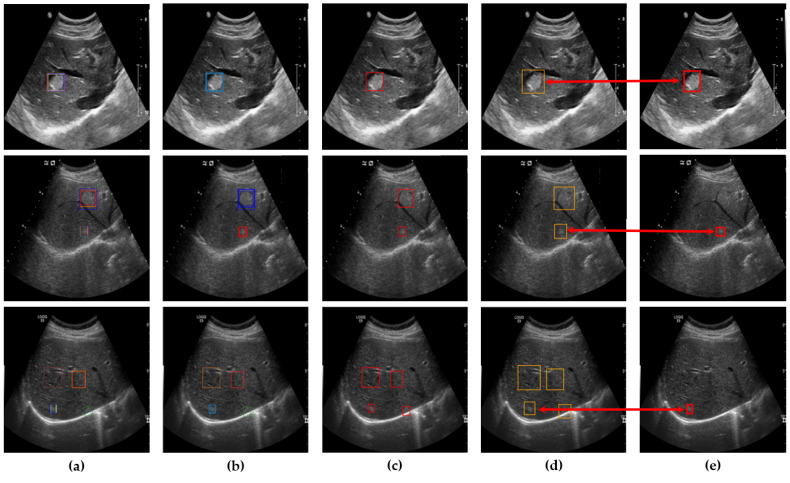
(**a**) An image with all bounding boxes detected by YOLOv8 overlaid, and the results of bounding box optimization through (**b**) clustering, (**c**) SM-WBF, and (**d**) padding. (**e**) Ground truth bounding box.

After clustering and outlier reduction, SM-WBF was applied to generate a single representative bounding box for each cluster. SM-WBF calculated weights for each bounding box by normalizing the mAP@0.5 values of the ten networks using the SoftMax function. These SoftMax weights were then used to combine the center coordinates and dimensions of the bounding boxes within each cluster through a weighted average approach. This method ensured that bounding boxes from higher-performing networks contributed more significantly to the result. Details of this process are illustrated in Figure 7.

### 3.2. Performance of Lesion Detection

To evaluate the accuracy of the bounding boxes generated through clustering, SM-WBF, and padding, we calculated the detection success rate on the test data. A detection was considered successful if at least one detected bounding box matched the ground truth. The matching criteria were based on the IoU between the detected box and the ground truth box with the same padding applied. If the IoU was 0.9 or higher, the boxes were deemed to match. Detailed results are presented in the table below.

Table 1 compares the results across four different scenarios, presenting mAP@0.5 scores, the number of correctly detected lesions in the test data, and the resulting accuracy. When a single YOLOv8 model was applied to the original US images without any preprocessing, the mAP@0.5 was 0.6128, and the model detected 101 lesions out of 180 test cases, achieving an accuracy of 56.11%. After applying our preprocessing steps to generate ten networks, clustering the bounding boxes, and performing SM-WBF, the method detected 172 out of 180 test cases, with an accuracy of 95.56%. In our evaluation using 180 test images, we compared the detection performance of YOLOv5, YOLOv8, and YOLOv11. YOLOv5 detected 167 lesions, YOLOv8 detected 172 lesions, and YOLOv11 detected 171 lesions, demonstrating that YOLOv8 achieved the best detection performance. It is important to note that YOLO has many versions, and the most suitable version varies depending on the dataset. The accuracy values presented here are not generalizable but are specific to our dataset.

Although the mAP@0.5 score was relatively low, this was because the clustering process preserved all potential bounding box regions, ensuring comprehensive lesion detection. However, the primary focus of our method is not achieving a higher mAP@0.5 but maximizing the capability to detect actual lesions, which demonstrates its performance was outstanding.

The bounding box optimization process contributed to enhancing the reliability and quality of the detection results by eliminating redundancies through clustering, refining outliers, and applying padding. The final bounding boxes encompassed the tumor contours without loss, providing a stable input for lesion classification models. This significantly improved the accuracy of the diagnostic support system.

### 3.3. Performance of Lesion Classification

**Figure 8:**
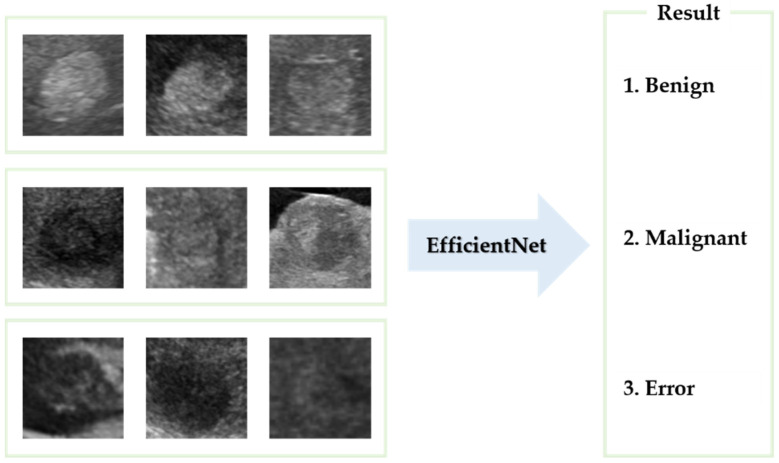
The lesions were classified into three classes using EfficientNet-b0.

The lesions identified through SM-WBF were classified using a CNN. Among various models, we selected EfficientNet-b0, known for its efficiency and performance, for comparison. As shown in Figure 8, the lesions were classified into three classes: benign, malignant, and error. For training, the image size was resized to 300 × 300, and the input size of EfficientNet-b0 was modified from its default 224 × 224 to 300 × 300. This adjustment was made to ensure accurate classification, as reducing the image size excessively could compromise the model’s performance.

The model was trained using the Adam optimizer with an initial learning rate of 0.001 and a momentum of 0.9. Additionally, the training data were shuffled at each epoch to introduce randomness among the data points, facilitating faster convergence towards the optimal solution.

If multiple boxes were detected in a single image, all boxes were classified. Boxes classified as error were ignored, and the output was determined as benign if only benign boxes were present. However, if even one box was classified as malignant, the output was set to malignant. Due to the nature of medical screening, where high sensitivity is more clinically significant than specificity, this approach was adopted to maximize practical utility for end users. Additionally, a 5-pixel padding was applied to enhance the visibility of lesion contours and facilitate comparisons between the internal color of the lesion and the surrounding liver tissue.

As shown in Table 2, the difference with and without padding was substantial. Without padding, the lesion contours resulting from clustering could become ambiguous, and comparing the color of the surrounding liver tissue with the lesion itself becomes nearly impossible. When padding was applied, these comparisons were feasible, leading to improved results. The detailed reasons for this will be discussed further in the discussion section.

Using a CNN, we performed inference on the 172 bounding boxes detected by SM-WBF. The dataset consisted of 114 benign classes, 58 malignant classes, and 48 error lesion boxes. The inference results are summarized in Table 3. Out of a total of 220 bounding boxes, 218 were correctly classified, achieving an accuracy of 99.09%, which is highly satisfactory. Moreover, the sensitivity, a critical metric for screening tests, was perfect 100%. Specificity was 98.24%, with two benign lesions misclassified as the error class. Importantly, as evidenced by the test results, no cases of misclassification of benign or malignant lesions due to error boxes occurred, alleviating a major concern.

The results in Table 3 highlight the robustness and reliability of the proposed neural network model as an effective screening tool for liver cancer detection. Additionally, the model can assist in identifying patients who require further examination, demonstrating its practical utility in clinical applications.

Rhyou et al. [37] proposed HCC-Net, which extracts lesions using YOLOv5 and applies wavelet transform. Each component was assigned ten different weight values, followed by inverse wavelet transform, generating a total of ten images. These outputs were concatenated into a new 10-channel dataset for classification, achieving a classifier sensitivity of 0.9732. In comparison, our proposed method achieved a sensitivity of 1.0000, demonstrating its superior ability to detect malignant lesions. This higher sensitivity indicates that our approach is more effective in minimizing false negatives, making it highly valuable for clinical screening applications where missing a potential malignancy could have serious consequences.

## 4. Discussion

Our methodology has three significant points. First, due to the inherent characteristics of ultrasound images, such as considerable speckle noise and variations in resolution depending on device settings, we employed various image processing filters to address these challenges. Using contrast enhancement filters like intensity adjustment and adaptive histogram equalization, as well as low-pass and high-pass filters, we generated 10 different frequency-domain images from a single ultrasound image. This data augmentation approach allowed us to create 10 networks, each with its own mAP@0.5 score, which were utilized in the SM-WBF process.

Second, we incorporated clustering and adapted the traditional WBF into SM-WBF to better suit our network. In conventional WBF, the confidence scores of bounding boxes are used as weights for fusion, effectively assigning greater importance to higher-confidence boxes. However, in our system, every bounding box contains critical information, and applying standard WBF risked losing valuable data. Additionally, we aimed to include all suspected lesions, necessitating the use of clustering. By employing clustering and SM-WBF, we achieved higher lesion detection accuracy while preserving critical information.

Lastly, we applied padding to retain richer features. The importance of comparing colors lies in its diagnostic significance. For screening where histopathological data are unavailable, color plays a vital role. Malignant lesions often exhibit distinctly different tones and patterns compared to surrounding normal tissues, whereas benign lesions generally appear uniform in color. Irregular internal color patterns suggest malignancy. Similarly, contours are critical; malignant lesions typically have irregular, spiculated, or serrated edges, reflecting their invasive nature. These lesions often display poorly defined boundaries due to infiltration into surrounding tissues, while benign lesions usually have smooth, rounded, or oval shapes with well-defined borders, often encapsulated. Such morphological differences based on contours and internal features are essential for distinguishing malignant from benign lesions. By applying padding, we ensured that these features were preserved, leading to significant performance improvements in detection.

However, the methodology also has limitations. The proposed approach involves data augmentation with 10 filters, inference on each US image using YOLOv8, clustering, outlier reduction, SM-WBF, and CNN-based classification, leading to high computational costs that are unsuitable for real-time operation. This trade-off was deemed acceptable for cancer screening, where accuracy is prioritized over real-time performance. As a future direction, model optimization for real-time applications will be explored. Furthermore, while our methodology demonstrates significant improvements in lesion detection, further validation on larger and more diverse datasets is necessary to confirm its robustness across different clinical environments. The variability in ultrasound imaging conditions, including differences in equipment, operators, and patient characteristics, may affect the generalizability of the proposed approach. To address this, future studies will focus on testing the model with external datasets from multiple institutions to assess its reliability and adaptability in real-world clinical applications. Additionally, setting a low threshold to capture all potential lesions led to the generation of unnecessary boxes, potentially creating redundant clusters during the clustering process. Further research will focus on refining the threshold values to minimize such occurrences.

## 5. Conclusions

By 2025, it is projected that over one million individuals will receive a liver cancer diagnosis annually. HCC constitutes approximately 75–85% of these cases. Fortunately, when detected at an early stage, HCC can be effectively managed before causing significant liver damage, emphasizing the critical need for efficient screening protocols for high-risk populations. However, fully automating the classification of liver cancer remains a significant challenge due to the inherent limitations of US imaging. These challenges include speckle noise, poor contrast between tumors and surrounding tissues, as well as the diverse morphology and echogenicity of lesions, all of which hinder their clinical applicability. Recently, various computer-aided diagnostic systems, including those leveraging deep learning techniques, have been developed to address these limitations and enhance hepatic lesion detection and classification.

This study presents “CSM-FusionNet”, a model achieving clinically acceptable accuracy through the targeted application of image processing filters, clustering, SM-WBF, and padding. Raw US images were collected from one of South Korea’s leading general hospitals, SMC. To overcome speckle noise inherent in ultrasound imaging and variations in resolution due to different settings, four types of filters were applied: intensity adjustment filter, adaptive histogram equalization filter, low-pass filter, and high-pass filter. These approaches not only reduced noise but also accounted for diverse resolutions, resulting in data augmentation that generated 10 US images from a single original image. Due to the limited nature of data acquisition for HCC, this method provided dual benefits by expanding the dataset. Each of the 10 US images was processed using the YOLOv8 model, producing 10 trained networks. The mAP@0.5 score of each network was measured and used to calculate weights for SM-WBF through SoftMax normalization.

Subsequently, bounding boxes were generated with a low threshold to identify multiple regions suspected of lesions, followed by clustering to segment these regions. Afterward, outliers were removed from each cluster, and SM-WBF was applied to merge multiple bounding boxes into a single one, reducing computational complexity. Padding was then added to the resulting bounding boxes to capture more features. The accuracy when using only original US images was 56.11%, which increased to 90.56% after applying clustering and WBF. Further application of SM-WBF elevated the accuracy to 95.56%, detecting lesions in 172 out of 180 test samples. Finally, classification was performed on the detected lesions, where padding also played a significant role. Without padding, the classification accuracy was 82.48%, while with padding, it increased to 97.58%, achieving 100% sensitivity—critical for computer aided diagnosis (CAD) systems.

Our technology holds significant potential to enhance the accuracy and efficiency of lesion detection and analysis using ultrasound imaging. By applying advanced fusion techniques like SM-WBF and clustering algorithms, it integrates information from multiple networks to deliver reliable diagnostic results. This enables clinicians to detect even minute lesions that might otherwise be overlooked, reduces diagnostic time, and supports better decision-making for personalized treatment. Furthermore, it enhances the efficiency of healthcare systems through automation and enables precise diagnostics even in resource-limited areas, improving healthcare equity. Ultimately, our technology underscores the importance of early diagnosis and preventative management, contributing to improved survival rates and enhanced quality of life for patients.

## Figures and Tables

**Table 1:** The mAP@0.5 scores for each network and the detection results on the test data.

Network		mAP@0.5	Detect	Accuracy
Original US image	with one YOLOv8	0.6128	101/180	0.5611
WBF	with 10 Network	0.7453	112/180	0.6222
WBF + clustering		0.5038	163/180	0.9056
SM-WBF + clustering		0.5172	172/180	0.9556

**Table 2:** The accuracy, sensitivity, specificity, and confusion matrix depending on the presence or absence of padding. Blue tones represent correct answers, while red tones represent incorrect answers.

	Without Padding	With Padding
Accuracy	0.8248	0.9758
Sensitivity	0.7903	1.0000
Specificity	0.8327	0.9703
Confusion Matrix	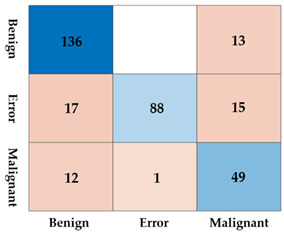	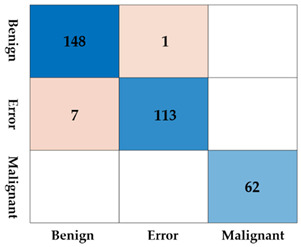

**Table 3:** The accuracy, sensitivity, specificity, and confusion matrix for 172 test datasets. Blue tones represent correct answers, while red tones represent incorrect answers.

Test with 172 Test Datasets			
Accuracy	0.9909	Confusion Matrix	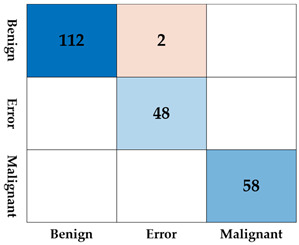
Sensitivity	1.000		
Specificity	0.9824		

## Data Availability

Data available on request due to restrictions.

## Abbreviations

The following abbreviations are used in this manuscript:

US	Ultrasound
HCC	Hepatocellular Carcinoma
SM-WBF	SoftMax-Weighted Box Fusion
SMC	Samsung Medical Center
DBSCAN	Density-Based Spatial Clustering of Applications with Noise
CNN	Convolutional Neural Network
YOLO	You Only Look Once
WBF	Weighted Box Fusion
IRB	Institutional Review Board
MoE	Mixture of Experts
mAP	Mean Accuracy Precision
IoU	Intersection of Union
CAD	Computer Aided Diagnosis
